# Animal models, treatment options, and biomaterials for female stress urinary incontinence

**DOI:** 10.3389/fbioe.2024.1414323

**Published:** 2024-08-29

**Authors:** Xiyang Tan, Guangzhi Li, Chenchen Li, Chenfan Kong, Huizhen Li, Song Wu

**Affiliations:** ^1^ Shenzhen Hospital, Shanghai University of Traditional Chinese Medicine, Shenzhen, China; ^2^ Department of Urology, The Third Affiliated Hospital of Shenzhen University, Shenzhen University, Shenzhen, China; ^3^ Department of Urology, The Affiliated South China Hospital of Shenzhen University, Shenzhen University, Shenzhen, China

**Keywords:** stress urinary incontinence, biomaterials, urethral bulking agents, tissueengineered repair materials, animal models

## Abstract

In the quest to tackle stress urinary incontinence (SUI), the synthesis of cutting-edge biomaterials and regenerative materials has emerged as a promising frontier. Briefly, animal models like vaginal distension and bilateral ovariectomy serve as crucial platforms for unraveling the intricacies of SUI, facilitating the evaluation of innovative treatments. The spotlight, however, shines on the development and application of novel biomaterials—ranging from urethral bulking agents to nano-gel composites—which aim to bolster urethral support and foster tissue regeneration. Furthermore, the exploration of stem cell therapies, particularly those derived from adipose tissues and urine, heralds a new era of regenerative medicine, offering potential for significant improvements in urinary function. This review encapsulates the progress in biomaterials and regenerative strategies, highlighting their pivotal role in advancing the treatment of SUI, thereby opening new avenues for effective and minimally invasive solutions.

## 1 Introduction

**FIGURE 1:**
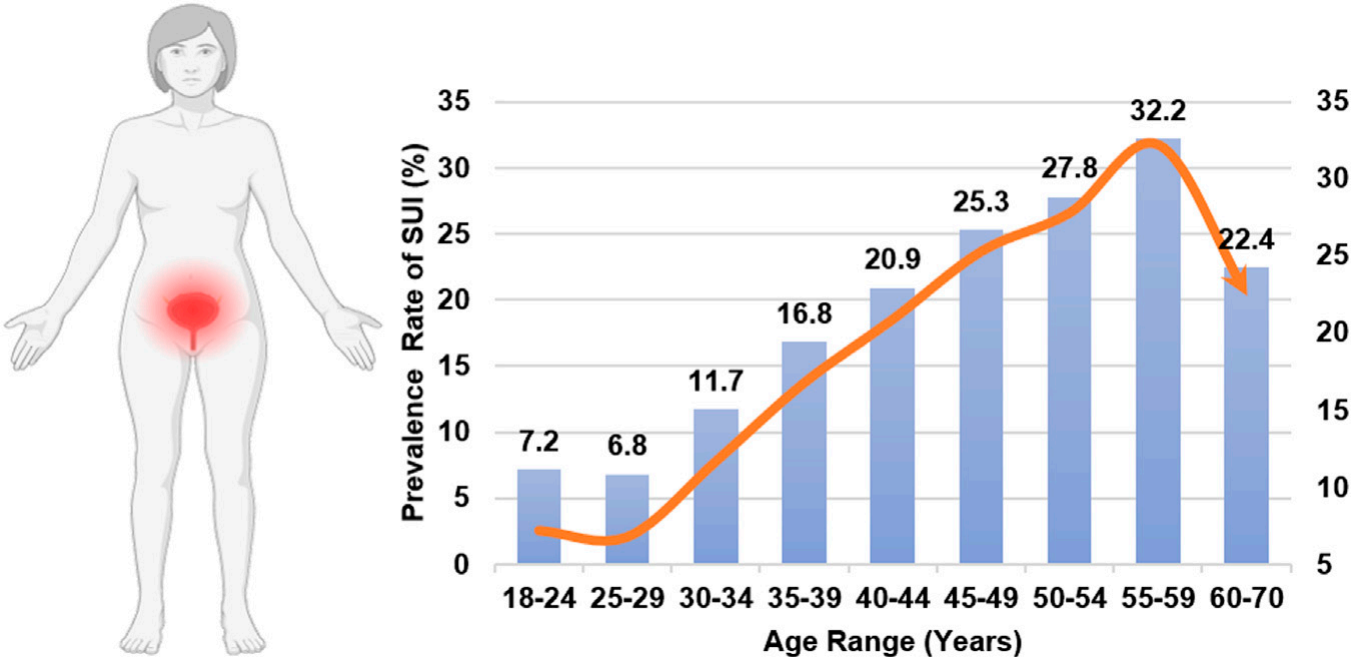
Distribution of SUI incidence rates according to age.

**FIGURE 2:**
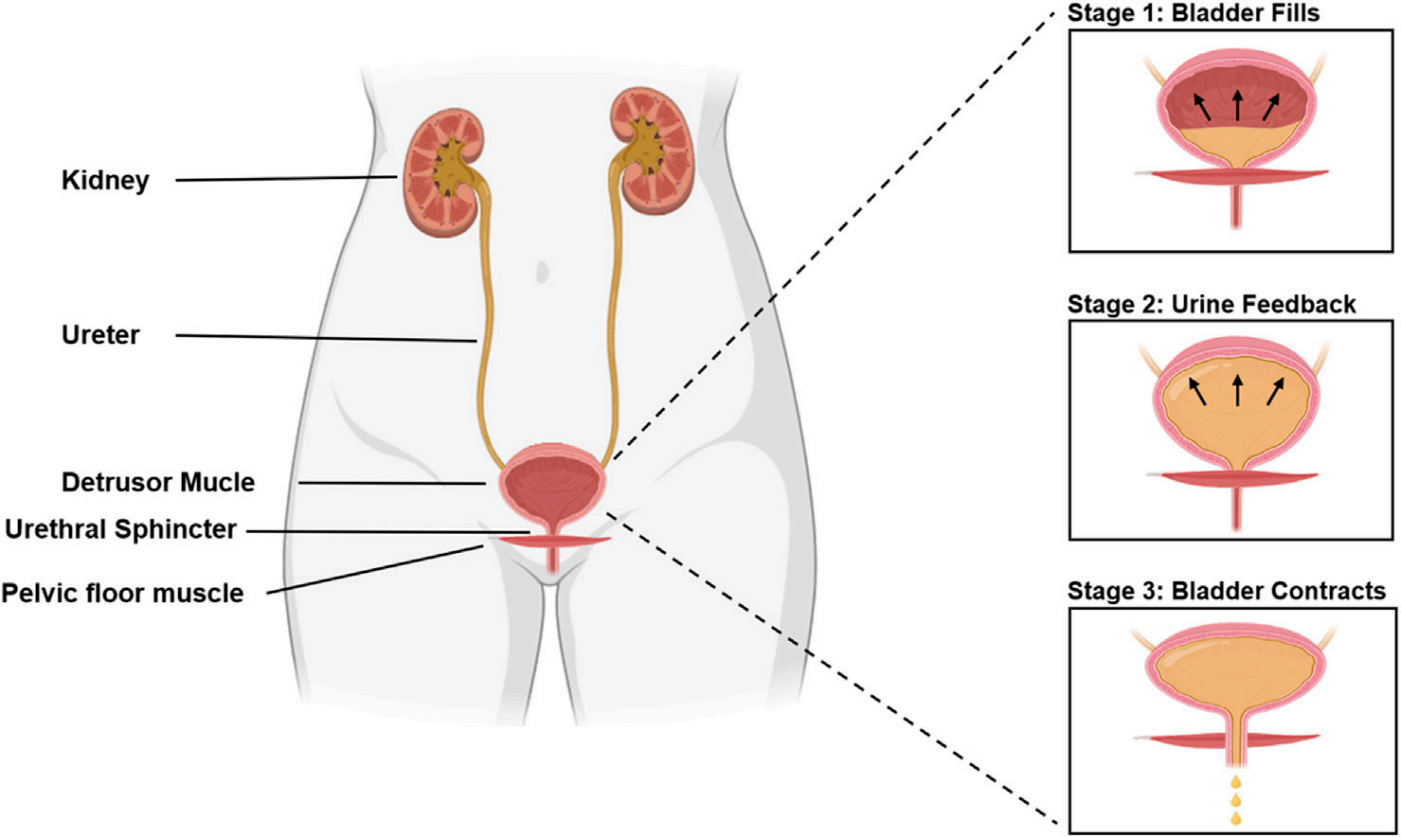
Urine excretion process in normal humans.

It is remarkable that SUI, a disorder impacting approximately one in three women and inducing distress on multiple fronts—physical, psychological, and social—does not garner more attention (Sims et al., 2022; Hu and Pierre, 2019). SUI significantly impacts normal lives, causing persistent anxiety and embarrassment, and imposes a significant economic burden on society (Hampel et al., 2004; Ford et al., 2017; Zhu et al., 2024). The incidence of urinary incontinence varies by age, as shown in Figure 1 (Gasquet et al., 2006). The occurrence of SUI is strongly associated with the bladder. Normally, urination is a voluntary process. When the bladder is full of urine, the pressure receptors on the bladder wall send signals to the brain, causing the sensation of the urge to urinate. The process of normal urination is depicted in Figure 2 (D'Ancona et al., 2019; Denisenko et al., 2021). SUI easily occurs when the anatomy of the pelvic nerves, urethra, and surrounding urethra area changes (Yang et al., 2022a; Ostrzenski, 2020). Research has confirmed that overweight and obesity are risk factors for SUI (Shang et al., 2023; Alsannan et al., 2024; Pang et al., 2023; Wang et al., 2023). The ideal treatment for SUI is not yet available. To enhance our understanding and treatment of urinary incontinence, this review aims to highlight the current advancements in the application of Urethral Bulking Agents (UBAs) and Tissue-engineered Repair Materials (TERMs) within the framework of disease models for SUI, underscoring the pivotal role of exploring biomaterials for therapeutic innovation.

## 2 Animal models in SUI

Animal models are essential in developing biomaterial therapies for SUI, enabling an understanding of the disease’s mechanisms and assessing therapies’ safety and effectiveness. These models replicate human SUI conditions, allowing researchers to test how biomaterials, such as UBAs and engineered scaffolds, repair urinary functions. They help optimize these materials for biocompatibility, degradation, and mechanical properties, ensuring suitability for human application. Additionally, animal studies are pivotal in devising minimally invasive techniques and guiding clinical trials, bridging the gap from laboratory research to clinical practice in SUI treatment. Disease models currently used for SUI are shown in Table 1.

**TABLE 1:** Representative SUI animal models.

Type	Model	Advantage	Shortage	Application
Induced	Vaginal distension, (Lin et al., 1998; Phull et al., 2011; Woo et al., 2009)	Simple operation, high survival rate	Poor durability	General SUI studies
	Bilateral Ovariectomy, (Kadekawa et al., 2020; Yoshida et al., 2007; Kitta et al., 2016)	Simple operation, high survival rate	Infection risk, poor stability	Estrogen deficiency, aging studies
	Nerve injury, (Damaser et al., 2003; Khorramirouz et al., 2016; Furuta et al., 2008; Pinar et al., 2022; Lee et al., 2004; Lee et al., 2003; Cruz et al., 2016)	Strong specificity, high stability	Infection risk, operating difficulty, severe trauma	Nerve injury-induced SUI studies
	Tissue destruction, (Hong et al., 2013; Kefer et al., 2009)	Long-term approach, high stability	Infection risk, severe trauma, high animal mortality	Surgical efficacy, muscle nerve regeneration
Spontaneous	ZF Rats, (Wang et al., 2017; Lee et al., 2017)	High stability, no need for post-molding	High cost	Transgenic research
	Gli2; Gli3^+/−Δ699/+^ Mice, (Yadav et al., 2023)	High stability, no need for post-molding	High cost	Transgenic research
	ERβ^−/−^ Mice, (Hamilton et al., 2017; Chen et al., 2014)	High stability, no need for post-molding	High cost	Estrogen receptor β studies
	Aged Rats, (Yanai-Inamura et al., 2019)	High stability, no need for post-molding	Poor stability	Aging studies

**FIGURE 3:**
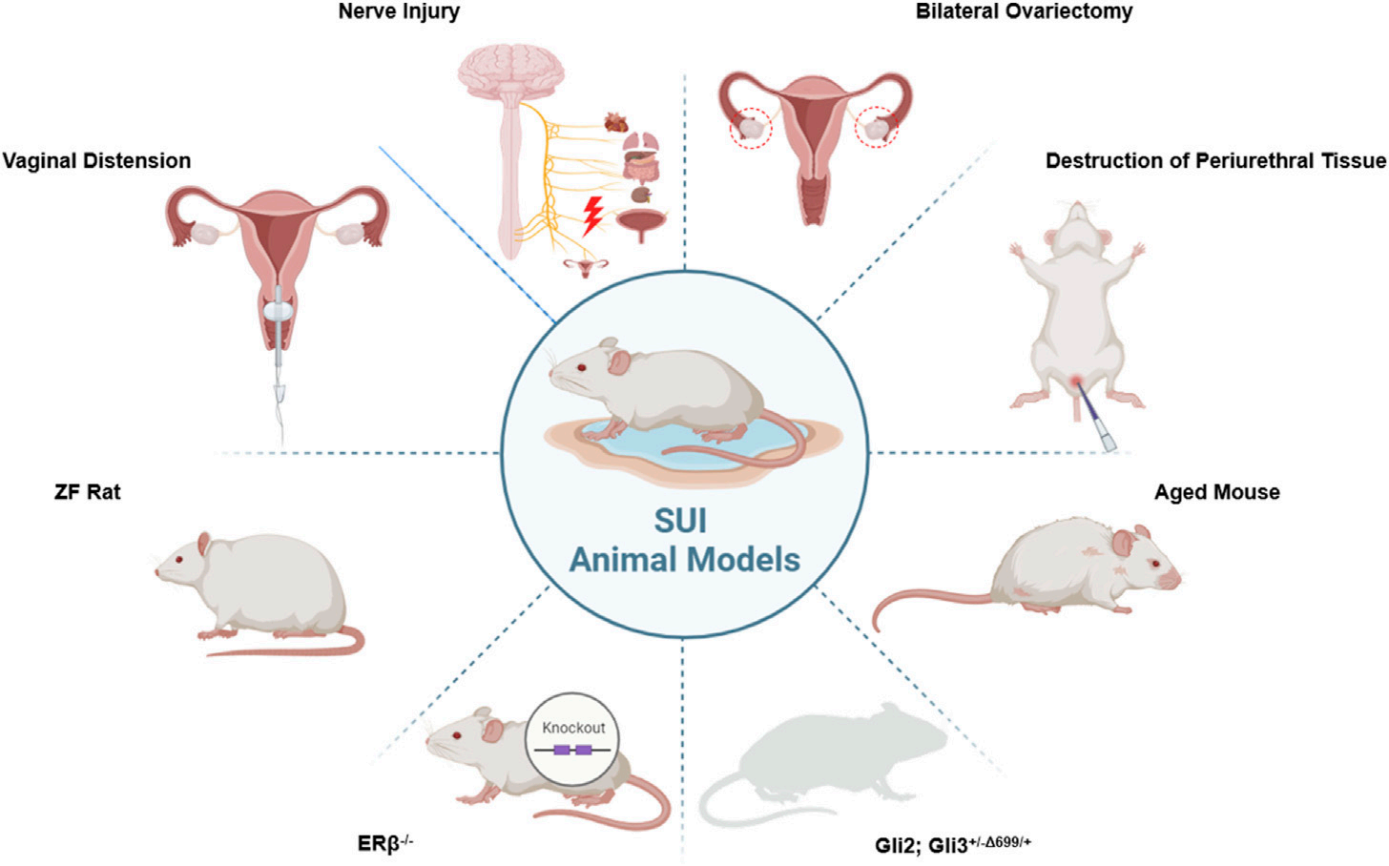
Animal models commonly used in research on SUI.

Animal models often involving small animals such as rats, rabbits, and mice, are divided into two primary types: Induction and Spontaneity. Each type mirrors particular facets of SUI and exhibits distinct characteristics. These modeling techniques are primarily employed in small animals like rats, rabbits, and mice. In addition, large animals including goats (Burdzinska et al., 2018), sheep (Khalifa et al., 2022), pigs (Burdzinska et al., 2018; Burdzińska et al., 2012; Kelp et al., 2017; Boissier et al., 2016), dogs (Kato et al., 1999; Ali-El-Dein and Ghoneim, 2001; Eberli et al., 2009), and cynomolgus monkeys (Badra et al., 2013a; Williams et al., 2016a; Badra et al., 2013b) can also serve as SUI models. The evaluation of animal models is crucial to the successful establishment of the model. Pathological sections serve as the standard for determining the successful establishment of an SUI model, but other methods can also be utilized to ascertain the success of animal sampling, such as Sneeze Testing, Manual LPP Testing, Vertical Tilt Table LPP Testing, and Electrical Stimulation (ES) LPP Testing (Jiang and Damaser, 2011). The animal models commonly used in current research for SUI are shown in Figure 3.

### 2.1 Induced animal model

#### 2.1.1 Vaginal distension

Vaginal distension (VD), or simulated birth trauma, is a method used to study the pathophysiology of SUI from childbirth and develop animal models. The procedure involves inserting a Foley catheter into the rat’s vagina, sealing the vaginal opening, and inflating the balloon with water or air. Within 4 h, edema is noticeable in the external urethral sphincter (EUS) and urethral/vaginal septum, with muscle disruption and fragmentation of the EUS occurring after 6 h. Inflammatory damage, indicated by polymorphonuclear leukocytes, peaks in the EUS after 4 h. Longer VD durations result in increased tissue edema, muscle damage, and changes in the urethra and vagina. After 2 weeks, the VD rats’ leak point pressure (LPP) significantly drops, with notable pathological differences persisting at 4 weeks in the urethra, pelvic ganglia, and levator muscle (Lin et al., 1998; Phull et al., 2011; Woo et al., 2009). Enhancing the model, a weight may be added to simulate traction injury (Wu et al., 2019). The specificity of EUS innervation and neurodegeneration suggests pudendal nerve vulnerability during VD (Damaser et al., 2003). While some believe model success may relate to the estrus cycle, studies indicate that the induction of reversible SUI in mice is not cycle-dependent (Huang et al., 2014).

#### 2.1.2 Bilateral ovariectomy (OVX)

Under isoflurane anesthesia, rats underwent a bilateral ovariectomy (OVX) through dorsolateral incisions, 2 cm below the last rib, followed by sutured wound closure. Six weeks later, OVX rats showed a marked decrease in continence, especially during sneezing, increased body weight, and reduced uterine weight, indicating successful estrogen depletion (Kadekawa et al., 2020). Previous studies have linked estrogen levels with body and uterine weights (Yoshida et al., 2007). Aging, compared to OVX-induced estrogen deficiency, more significantly affects baseline urethral function over reflex activity of striated muscles, with estrogen deficiency further impairing continence reflexes. Thus, aging and estrogen deficiency synergistically impact urethral function and continence mechanisms, contributing to SUI development (Kitta et al., 2016).

#### 2.1.3 Nerve injury

The pudendal nerve, located in the ischiorectal fossa, was thoroughly crushed twice, for a duration of 30 s on each side, near the obturator nerve’s branch point using a Castroviejo needle holder. The immediate visualization of the nerve injury was facilitated by the transparency of the nerve sheet at the crush location. This technique is termed pudendal nerve crush (PNC). Following LPP testing, it was observed that the control group’s LPP value was significantly higher than that of the pudendal nerve compression group (Damaser et al., 2003). Bilateral pudendal nerve resection at the level of the lumbosacral trunk anastomosis in rats constitutes the procedure known as pudendal nerve transection (PNT). HE and IHC staining revealed irreversible loss of striated muscle mass in the sphincter region and an increase in collagen deposition indicative of muscle atrophy. Significant decreases in LPP measurements were also observed following bilateral PNT (Khorramirouz et al., 2016). The underlying principle of pudendal nerve ligation (PNL) mirrors that of pudendal neurotomy. After nerve blockade, sensory loss in the innervated area and muscle loss of contractility occurs, leading to SUI (Furuta et al., 2008). Bilateral pelvic nerve injury (PNI) (Pinar et al., 2022; Lee et al., 2004), sciatic nerve transection (Lee et al., 2003), and impairment of the dorsal nerve of the clitoris (Cruz et al., 2016) can also induce SUI in rats.

#### 2.1.4 Destruction of periurethral tissue

The content of smooth muscle in the cauterized group was significantly reduced, making vascular expression almost imperceptible due to the destruction of the tissue around the urethra through electrocautery (Hong et al., 2013). This model serves as a viable animal model for studying angiogenesis, neurogenic, and myogenic injuries. After the transection of the pubo-urethral ligament, the LPP value showed a significant decrease, mirroring the effects observed in the modeling method of nerve transection (Kefer et al., 2009).

#### 2.1.5 Other methods

The nerve anesthetic botulinum-A toxin, a neuromuscular blocking agent, paralyzes muscle nerves and blocks the transmission of information between motor nerves and muscles. Following the administration of botulinum-A toxin around the urethra in rats, an increase in urinary output and muscle tissue atrophy were observed (Bandyopadhyay et al., 2014). Spinal cord injury (SCI) frequently results in neurogenic detrusor overactivity (NDO) as a result of the sprouting of sensory afferents on the lumbosacral spinal cord. NDO is characterized by a high frequency of voiding contractions and an increased intravesical pressure, potentially leading to SUI. Interestingly, injections of botulinum-toxin A into the bladder wall constitute an effective option to manage NDO (Coelho et al., 2016). To produce the retroflexed bladder, the bladder was surgically attached posteriorly to the psoas muscle. In rats with a retroflexed bladder, a marked reduction in both the urethral pressure reaction and sneeze-induced LPP was observed (Kawamorita et al., 2010). Inducing urinary bladder overactivity and urinary incontinence in mice through the diuretic Furosemide is another method (Saporito et al., 2016). SUI is prevalent in individuals with Parkinson’s disease (PD). Based on these clinical observations, a comorbid disease model of PD and SUI was developed. A rat model was induced through the injection of 6-hydroxydopamine. Depletion of dopamine significantly impairs the active urethral closure mechanism (Ouchi et al., 2019). To ensure the stability and suitability of the experimental animal model, combining multiple modeling methods is often advantageous. Examples include combining VD with OVX (Li et al., 2022; Li et al., 2016), PNT (Ni et al., 2018), and PNC (Balog et al., 2023).

### 2.2 Spontaneous animal models

The main reason for the popularity of spontaneous animal models lies in their ability to minimize artificial modeling factors, thereby more closely resembling naturally occurring human diseases. Among experimental animals exhibiting genetic obesity, Zucker fatty (ZF) rats represent the most widely utilized models. This condition is attributed to the recessive gene fa, resulting from a mutation in the Fa gene. The model includes two sublines: lean and obese types. Obese ZF rats exhibit characteristics remarkably similar to human obesity and the early stages of type II diabetes, including insulin resistance, an abnormal glucose tolerance test, and a normal fasting glucose. Furthermore, it presents a series of obesity-related complications, including high blood lipids and reduced reproductive capacity. Obesity impairs urethral sphincter function through IMCL deposition, leading to atrophy and distortion of urethral striated muscle. Therefore, ZF rats could serve as a consistent and reliable animal model for studying obesity-associated SUI (Wang et al., 2017). Interestingly, ZF rats exposed to single pulse electric field stimulation (EFS) during organ bathing experiments may exhibit SUI and compromised urethral sphincter contractility (Lee et al., 2017).

The double mutant GLI family zinc finger Gli2; Gli3^+/−Δ699/+^ murine model of SUI has recently been developed as a reliable model. Histological changes in the bladder neck and urethra can directly lead to SUI, with prostate hypoplasia being a possible additional factor. This may be related to premature termination of the spinal cord (Yadav et al., 2023).

Estrogen exerts its effects through two distinct nuclear receptors: estrogen receptor alpha (ERα) and estrogen receptor beta (ERβ). ERβ is believed to be associated with the onset of SUI. The primary site of ERβ expression is in the granulosa cells of the ovary. A significant decrease in LPP and maximum urethral closure pressure (MUCP) values in the ERβ^−/−^ group, compared to the ERβ^+^ group, indicates that ERβ gene knockout plays a critical role in the development of SUI (Hamilton et al., 2017; Chen et al., 2014). The pathological mechanism of this approach is akin to that of OVX in rats, which influences estrogen levels.

In clinical practice, the risk of SUI escalates with age (Pearlman and Kreder, 2020). These clinical characteristics constitute the primary rationale for employing elderly rats in the SUI model. Compared to young (3 months) and middle-aged (12 months) rats, aged rats (24 months) exhibited a significant decrease in LPP value, an increase in the area of connective tissue in the EUS layer, a significant decrease in the number of EUS fibers, and an increase in the cross-sectional area of EUS fibers (Yanai-Inamura et al., 2019).

## 3 The current therapies

SUI is widely recognized as a global public health issue, with effective management significantly enhancing patients’ quality of life. Currently, a variety of therapeutic options are available. Clinicians with relevant experience should determine the appropriate intervention—conservative or surgical—based on the severity of the patient’s symptoms.

Non-surgical measures are generally recommended as the initial approach for managing SUI. Pharmacological treatments, including anti-muscarinic agents, antidepressants, estrogen, botulinum toxin, and α-adrenergic agonists, can increase urethral sphincter pressure or decrease bladder activity. However, when conservative measures fail to provide the desired therapeutic outcomes, surgical intervention becomes necessary.

In recent years, there have been significant advancements in surgical techniques for SUI. Options now include bladder neck injections, midurethral slings, vaginal suspensions, and autologous fascia slings (Nightingale, 2020). Table 2 summarizes the current methods used to treat SUI.

**TABLE 2:** Comparison of conservative and surgical treatment.

Comparison aspect	Conservative management	Surgical management
Treatment Methods	Acupuncture and Moxibustion, Pelvic Floor Muscle Training (PFMT), Yoga, Lifestyle Modifications, Medications (anti-muscarinics, β-adrenergic agonists, duloxetine, estrogen)	Midurethral sling, Urethral Injection, Uratape, Colposuspension, Autologous Fascial Sling, Laparoscopic Vaginal Suspension
Effectiveness	Can improve symptoms but may not fully resolve SUI	Generally higher success rates, especially for severe cases
Invasiveness	Non-invasive or minimally invasive	Invasive, involves surgery
Risks and Complications	Low risk, mainly related to side effects of medications. Research methodology improvements needed (randomization, blinding, treatment interpretation)	Risks of surgical complications (e.g., bladder, intestinal, vascular injuries)
Recovery Time	No significant recovery time needed	Requires recovery period; minimally invasive options have shorter recovery times
Suitability	Suitable for mild to moderate SUI or as first-line treatment	Suitable for moderate to severe SUI or when conservative treatments fail
Long-term Outcomes	May require ongoing management and follow-up. PFMT long-term benefits with short-term follow-up	Long-term improvement with many procedures showing high cure rates
Cost	Generally lower cost	Higher cost due to surgical procedures
Evidence Level	Mixed evidence, with some treatments needing further research validation (e.g., acupuncture). High-level evidence for PFMT	Extensive research supports effectiveness, particularly for MUS

### 3.1 Conservative management

Although surgery can reduce leakage to some extent, it cannot fully resolve SUI (Nygaard and Norton, 2022). Other essential interventions include acupuncture, moxibustion, pelvic floor muscle training (PFMT), yoga, and lifestyle modifications.

Acupuncture and moxibustion, rooted in ancient Eastern traditions, treat diseases by stimulating acupoints and require consideration of individual differences for syndrome differentiation. While these methods have shown improvements in urine leakage (Liu et al., 2017), meta-analyses indicate that they are not superior to drug treatments (Wang et al., 2013). Improvements in research methodologies, including randomization, blinding, treatment interpretation, and acupuncturist certification, are needed (Yang et al., 2022b). Current statistical analysis methods in randomized controlled trials are also often inadequate (Liu et al., 2022).

PFMT for female SUI has received an A-level evidence rating based on numerous RCT meta-analyses (Dumoulin et al., 2011). Long-term follow-up can maintain the benefits of PFMT without additional training incentives. Significant differences exist in the quality of interventions between short-term and long-term PFMT studies, yet short-term results can be preserved during long-term follow-up (Bø and Hilde, 2013). The mechanisms include enhancing pelvic floor muscle strength, improving timing awareness, and strengthening core muscles (Sheng et al., 2022).

Yoga, a psychosomatic therapy, has been effective in improving urinary incontinence and can be combined with PFMT (Tenfelde and Janusek, 2014; Kim et al., 2015). Lifestyle modifications, such as weight reduction, can also alleviate urinary incontinence symptoms associated with obesity (Kerdraon and Denys, 2009). Massage treatment has shown significant reductions in urine leakage, with complete symptom alleviation after 1 month of discontinuing therapy in some cases (Kassolik et al., 2013).

Medical management is a crucial aspect of SUI treatment. Anti-muscarinic drugs, the first-line treatment for overactive bladder, include oxybutynin, propiverine, tolterodine, solifenacin, darifenacin, trospium, imidafenacin, and fesoterodine (Swinburn et al., 2011; Yamada et al., 2018). These drugs can cause side effects like dry mouth, blurred vision, and constipation, depending on their specificity for bladder muscarinic receptors. When anti-muscarinic drugs are unsuitable, β-adrenergic receptor agonists like mirabegron and solabegron are effective alternatives (Mostafaei et al., 2022; Ellsworth, 2012). Meta-analyses have shown their effectiveness comparable to anti-muscarinic drugs (Kelleher et al., 2018; Ohlstein et al., 2012).

Pharmacological research suggests lower urinary tract activity is centrally regulated by 5-HT and NE receptor agonists and antagonists. Duloxetine, a 5-hydroxytryptamine/norepinephrine reuptake inhibitor, treats SUI by inhibiting serotonin and norepinephrine reuptake at the presynaptic neuron in Onuf’s nucleus of the sacral spinal cord (Bitter et al., 2011; Jost and Marsalek, 2004). Research supports duloxetine as a viable treatment option for female SUI (Deepak et al., 2011; Mariappan et al., 2007; Basu and Duckett, 2009).

Hormone therapy is commonly used for lower urinary tract symptoms. Estrogen can increase urethral pressure, significantly improving or curing SUI in many cases (Elia and Bergman, 1993). However, evidence for estrogen supplementation’s effectiveness in improving urinary incontinence remains insufficient (Quinn and Domoney, 2009). In patients with bladder muscle overactivity and urinary incontinence due to nervous system disease, botulinum toxin injections have improved symptoms and bladder function without side effects (Kennelly et al., 2022).

### 3.2 Surgical management

When general management proves ineffective, surgical intervention becomes necessary. The advent of minimally invasive, retropubic, synthetic midurethral sling operations has revolutionized SUI surgery, surpassing traditional procedures like colposuspension and autologous fascial slings (Giarenis and Cardozo, 2014). The success of these surgeries depends on the surgeon’s experience, but risks include bladder, intestinal, or vascular damage via the obturator approach.

Surgical options for SUI include open abdominal retrovaginal suspension, anterior vaginal repair, suburethral suspension, bladder neck pin suspension, periurethral or transurethral injection of fillers, artificial urethral sphincters, and laparoscopic vaginal suspension (Kirchin et al., 2017). Among these, retrovaginal suspension, slings, and urethral dilation injections are the most commonly employed techniques. Urethral mid-suspension is a preferred minimally invasive procedure due to its low complication rate and favorable prognosis, while urethral dilation injections are safer for high-risk patients (Umoh and Arya, 2012).

The Uratape procedure, which involves tension-free tape insertion beneath the urethra, and transvaginal tape slings are commonly used techniques. Despite their effectiveness, complications such as bladder, intestinal, and vascular injuries can occur (Delorme et al., 2004; Reisenauer et al., 2006). The development of the midurethral sling (MUS) has been crucial, with various slings available that are inserted at the midurethral level either retropubically or transobturatorily (Rapp et al., 2009; Kane and Nager, 2008). MUS procedures typically involve minimal incisions, quick recovery, and high cure rates with minimal complications. Long-term outcomes are generally positive, with patients resuming normal activities shortly after surgery (Ford et al., 2017). Traditional slings are as effective as minimally invasive slings but have a higher incidence of adverse reactions. They exhibit a similar cure rate to open posterior pubic vaginal suspension, though long-term adverse events remain unclear (Rehman et al., 2017).

Urethral injections (periurethral/intraurethral) are a well-established minimally invasive treatment for uncomplicated SUI, particularly in patients unresponsive to conservative management or those contraindicated for drug treatment (Chapple et al., 2005). Various agents, such as autologous fat, carbon beads, calcium hydroxyapatite (CaHA), ethylene vinyl alcohol copolymer (EVOH), glutaraldehyde cross-linked bovine collagen, porcine dermal implants, polytetrafluoroethylene, and silicone particles, can be used as fillers. Despite insufficient evidence to fully guide clinical practice, urethral fillers may offer a cost-effective alternative to other surgical options (Kirchin et al., 2017).

## 4 The ideal material

The ideal biomaterial would be readily available, cost-effective, chemically and physically inert, sterile, non-carcinogenic, mechanically robust, resistant to alteration by bodily tissues, and carry a low risk of infection and rejection. After healing from incontinence surgery, the graft should restore the pelvis to its natural structure and function, mirroring the durability of autologous tissue. Furthermore, the material must remain in place long enough to be integrated by the host tissue. To minimize impact on the surrounding area during surgery, the biomaterial should be supple, able to withstand mechanical stress and shrinking, and flexible (Sangster and Morley, 2010).

Synthetic biomaterials have potential applications in treating urinary incontinence. Synthetic materials often address urinary incontinence problems through implants, injections, films, or tablets. They increase the ability of the urethra to close by strengthening the urethral sphincter or supporting the urethral tissue, thereby reducing urine leakage.

## 5 Urethral bulking agents (UBAs)

The primary mechanism by which UBAs work involves the injection of these materials around the urethra, leading to an increase in urethral volume. This augmentation helps to coapt the urethral lumen, enhancing the closure pressure at rest and improving the functional length of the urethra, which is crucial for maintaining continence.

### 5.1 Plasmid DNA-loaded injectable agent

The researcher prepared pDNA (encoding for bFGF) complex-loaded poly (dl-lactic-co-glycolic acid) (PLGA)/Pluronic F127 mixture solution dispersed with polycaprolactone (PCL) microspheres as an injectable bioactive bulking agent (Choi et al., 2013). The mixture solution was designed to solidify at the injured site upon contact with water or body fluid, thus enabling stable deposition of PCL microspheres in the solidified PLGA matrix to maintain its initial volume without migration. The researcher anticipated that the prepared bulking agent could provide a passive bulking effect (by PCL microspheres) and allow prolonged stimulation of the defect tissues around the urethra (through long-term production of bFGF from the cells transfected by continuously released pDNA) for the effective treatment of SUI.

### 5.2 Macro/nano-gel composite

Nanogels present several advantages for drug delivery, including high biocompatibility from their natural or synthetic polymer composition, which reduces immune reactions and toxicity. They enable controlled drug release, with their network structure allowing for modulated release rates, which improves efficacy and reduces side effects. Additionally, encapsulated drugs in nanogels are protected, increasing their stability. Surface modifications of nanogels enhance targeting to specific cells, making them an effective drug delivery system.

**FIGURE 4:**
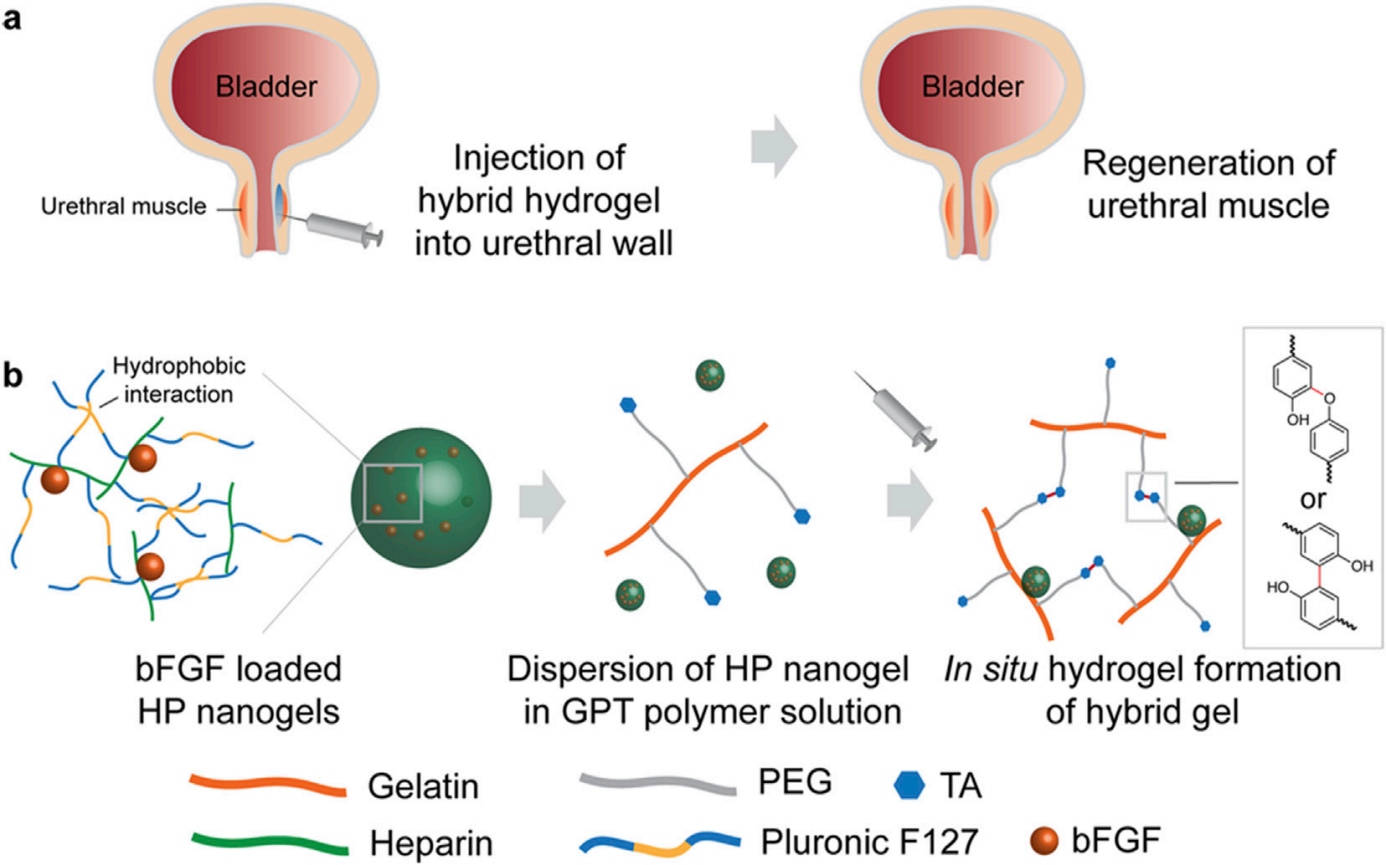
Regeneration approach and construction of hybrid macro/nanogel composites. **(A)** Diagram illustrating urethral muscle regeneration utilizing an injectable, bioactive bulking hydrogel. **(B)** Formation of bFGF-loaded HP nanogels via hydrophobic interactions. Reprinted with permission from ref. (Park et al., 2014). Copyright 2014, ACS.

Researchers have discovered a specific macro/nanogel type useable as an injectable, bioactive bulking agent for treating incontinence. It comprises *in situ* generated gelatin-based macrogels and self-assembling heparin-based nanogels. These hybrid hydrogels are produced via an enzymatic process involving hydrogen peroxide and horseradish peroxidase. To create a hybrid gel matrix capable of continuously releasing growth factor (GF) for up to 28 days, a gelatin gel matrix was mixed with a GF-loaded heparin nanogel. Furthermore, these hydrogel composites promote the regeneration of urethral muscle tissue around the urethral wall, aiding in the restoration of biological function upon *in vivo* injection (Park et al., 2014). The method for the regeneration and creation of composites combining macro and nanogel is illustrated in Figure 4.

### 5.3 Liquid crystal elastomer based dynamic device for urethral support

This is a novel device of liquid crystal elastomers (LCE) inspired by static slings that non-invasively adjust their tension by changing shape in response to temperature increases induced by transcutaneous infrared light, thereby reducing urethral resistance and improving urination, studies have shown that the LCE-CB device can treat SUI by increasing the urethral resistance to leakage under the application of abdominal pressure (Tasmim et al., 2023).

### 5.4 CAD/CAM collagen scaffolds for tissue engineering

**FIGURE 5:**
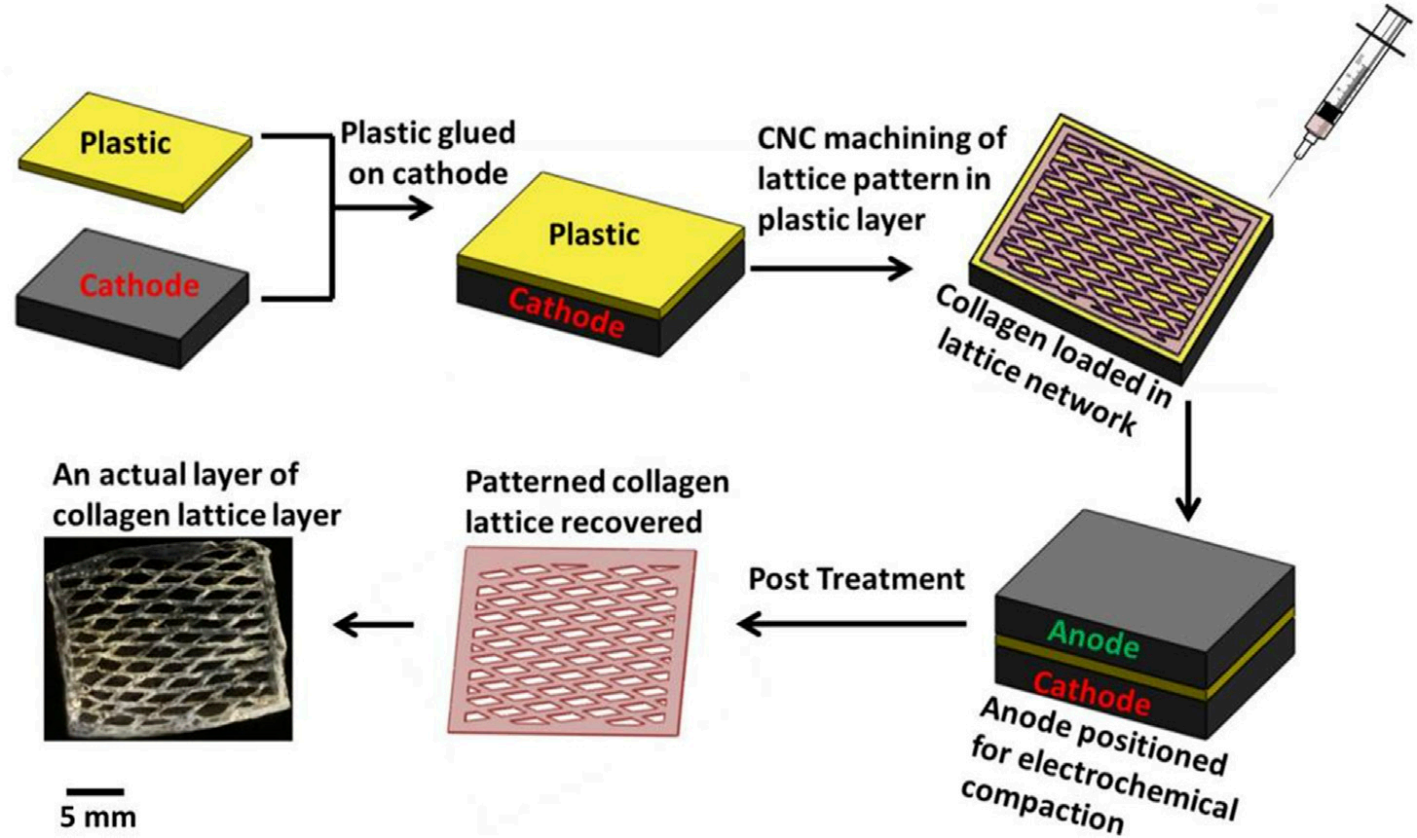
Process of fabricating individual patterned layer. Reprinted with permission from ref. (Islam et al., 2015). Copyright 2015, IOP Publishing.

The present research has established a method utilizing CAD/CAM technology for the creation of a scaffold composed entirely of collagen, characterized by its high porosity and patterned structure. The dense packing and alignment of the collagen molecules have endowed the scaffold with significant mechanical strength. It has been indicated that adjustments to the structure’s number of layers and its shape could allow for the tuning of its mechanical characteristics to suit various applications in tissue engineering, including SUI (Islam et al., 2015). The scaffold is shown in Figure 5.

### 5.5 Other commercial UBAs

#### 5.5.1 Ethylene vinyl alcohol (Tegress™)

Ethylene vinyl alcohol (EVA) copolymer serves as a permanent filler. It exhibits good biocompatibility and a low propensity for allergic reactions (Kuhn et al., 2008). However, a follow-up study involving 20 patients revealed that a significant proportion experienced serious complications. These complications included surgical-related issues and urinary tract erosion (Hurtado et al., 2007). A subsequent study also reported similar complications (Hurtado et al., 2008). Due to these studies and growing reports of complications associated with Tegress™, the manufacturer voluntarily removed the bulking agent from the market in December 2007 (Mukkamala et al., 2013).

#### 5.5.2 Dextranomer/hyaluronic acid copolymer (Deflux™, Zuidex™)

Dextranomer, a highly hydrophilic dextran polymer solubilized in a base of non-animal stabilized hyaluronic acid, has received approval as an injectable agent for childhood vesicoureteric reflux (Deflux™) and, in Europe, for women with SUI (Zuidex™) (Lightner et al., 2010). A study comparing Deflux™ and Teflon™ as bladder neck injections in children found that neither the number of injections nor the type of filling agent impacted the outcome: short-term improvements were observed, but long-term effects were lacking]. Researchers have concluded that bladder neck injections of filling agents are generally ineffective for treating incontinence (Dyer et al., 2007). Another dextranomer/hyaluronic acid copolymer, Zuidex™, used as a urinary filler, has shown through clinical evidence to significantly improve patients’ subjective quality of life and objective incontinence measurements post-treatment (van Kerrebroeck et al., 2004). However, reports of side effects have emerged, including the formation of uretrovaginal fibrosis associated with “sterile abscission” post-treatment (Hilton, 2009).

#### 5.5.3 Glutaraldehyde rross-linked collagen (Contigen™)

Glutaraldehyde cross-linked (GAX) collagen, a sterile injection gel, is composed of highly purified bovine dermal collagen, cross-linked with 0.0075% glutaraldehyde, and suspended in physiological saline solution with 0.3% lidocaine. After receiving FDA approval in 1985, it has been primarily used as an intradermal therapy for correcting soft tissue contouring abnormalities. Subsequently, its applications expanded to include use as a urethral filler for urinary incontinence. While its efficacy is supported by clinical evidence (Stricker and Haylen, 1993; Shortliffe et al., 1989), some studies question its long-term effectiveness (Sundaram et al., 1997). Currently, reported adverse reactions include local injection calcification (Knudson et al., 2006), hematuria, urinary retention, urethral mucosal prolapse, among others (Stothers et al., 1998; Harris et al., 1998). In 2012, GAX was officially withdrawn from the European market (Lüttmann et al., 2019).

#### 5.5.4 Calcium hydroxylapatite (Coaptite™)

Hydroxyapatite calcium (CaHA, Coapatite™) consists of spherical CaHA particles with diameters ranging from 75 to 125 microns and was approved by the US FDA in 2005 (Csuka et al., 2023). Evidence suggests that hydroxyapatite particles may serve as effective fillers for treating stress incontinence (Mayer et al., 2001; Griffin et al., 2016). A 12-month clinical study comparing CaHA (Coapatite) with Contigen™ found that both CaHA and collagen were well tolerated, with no systemic adverse events observed for either product (Mayer et al., 2007).

#### 5.5.5 Carbon coated beads (Durasphere™)

Durasphere™, an injectable bulking agent featuring carbon-coated beads, has FDA approval for treating SUI. Its confirmed and potential benefits include non-immunogenicity, tissue non-responsiveness, efficacy with minimal injection volume, and durability (Pannek et al., 2001; Madjar et al., 2003). Unfortunately, similar to Contigen™, concerns exist regarding Durasphere’s long-term efficacy (Chrouser et al., 2004). Carbon-coated beads are not superior to current alternative filler biomaterials due to consistent particle migration and limited success (Pannek et al., 2001).

#### 5.5.6 Polytetrafluoroethylene (Teflon™)

Polytetrafluoroethylene (PTFE) is a type of plastic fluoropolymer material (Becmeur et al., 1990). Previously, Teflon™ was utilized as a urethral filler for treating urinary incontinence (Kaufman et al., 1984). This method involves injecting granules into the tissues surrounding the urethra to increase closing pressure and alleviate urinary incontinence symptoms. However, research has identified potential problems and complications associated with this treatment method. One of the primary concerns involves potential organizational reactions and side effects. Long-term use of this filler material can result in complications, including tissue inflammation, infection, foreign body reactions, and urethral stricture. Additionally, the filler material may shift or disperse within the surrounding urethral tissues, resulting in unstable treatment outcomes (McKinney et al., 1995; Kiilholma et al., 1993; Martínez-Piñeiro et al., 1989; Kiilholma and Mäkinen, 1991).

#### 5.5.7 Polydimethylsiloxane (Urolastic™) and cross-linked polydimethylsiloxane (Macroplastique™)

Polydimethylsiloxane (Urolastic™), an organic polymer containing silicon, is widely used in medical and biological fields. It has been approved by the Food and Drug Administration in 2006 as a treatment option for urinary incontinence (Serati et al., 2023). Injecting polydimethylsiloxane into the tissue surrounding the urethra increases its support and function. The treatment aims to enhance urethral sphincter function and reduce urinary incontinence symptoms. Three-year cohort research suggests Macroplastique™ as a viable SUI treatment alternative, offering long-lasting results and a low incidence of side effects (Serati et al., 2019). A multicenter, randomized, controlled, single-blind study shows cross-linked polydimethylsiloxane to be more effective than Contigen™ (Ghoniem et al., 2009).

#### 5.5.8 Pig collagen protein (Permacol™)

A bioactive, injectable bulking agent comprising Permacol™ and recombinant insulin-like growth factor-1 conjugated to fibrin micro-beads (fib_rIGF-1), known for its bulk stability and ability to induce muscle regeneration. Research has confirmed its ability to promote angiogenesis at the injection site and to foster smooth muscle tissue formation (Vardar et al., 2019). A prospective randomized trial revealed Permacol’s superiority over Macroplastique™ in treatment efficacy (Bano et al., 2005). Additionally, it significantly enhances function and quality of life in cases of constipation, fecal incontinence, and prolapse, and also ameliorates the ecological symptoms of the urinary system (McLean et al., 2018).

#### 5.5.9 Biomimetic synthetic materials (Regensling™)

The aim of Regensling™, a synthetic sling device featuring biomimetic construction, is to provide long-term mechanical support while minimizing the risk of complications. Regensling™ exhibited a milder inflammatory response compared to the control material and was enveloped in a thin layer of fibrous tissue, showing good compliance. Throughout the testing, Regensling™ demonstrated consistent strength, with a notable upward trend (Lai et al., 2017).

#### 5.5.10 Polyacrylamide hydrogel (Bulkamid^®^, Aquamid™)

Polyacrylamide hydrogel (PAHG) includes Bulkamid^®^ and Aquamid™. Research has confirmed that Bulkamid^®^ is at least as effective as Contigen™. Bulkamid^®^ has shown positive, long-lasting effects on stress incontinence with minimal risk of significant side effects. With Contigen™ no longer on the market, Bulkamid^®^ emerges as a promising treatment option for women with stress incontinence due to its straightforward bulking method (Sokol et al., 2014; Brosche et al., 2021). Overall, 21% of women did not respond adequately to PAHG (Bulkamid^®^) injection therapy. The therapy had few moderate short-term adverse effects and no significant long-term adverse events (Hoe et al., 2022). For PAHG (Aquamid™), treatment outcomes are similar, albeit with some adverse events like urinary retention and urinary tract infections. Similar results were observed with PAHG (Aquamid™) therapy, though side effects such as bladder retention and urinary tract infections were reported (Lose et al., 2006). Commercial UBAs are shown in Table 3.

**TABLE 3:** Commercial UBAs for SUI.

Ref.	Year	Object	Biomaterial	Key point
Kuhn et al. (2008)	2007	Human	Ethylene Vinyl Alcohol (Tegress™)	Permanent filling material, good biocompatibility, not prone to allergies
Lightner et al. (2010), Dyer et al. (2007)	2007 2010	Human	Dextranomer/hyaluronic acid copolymer (Deflux™, Zuidex™)	Poor long-term effect
Sundaram et al. (1997)	1997	Human	Glutaraldehyde Rross-Linked collagen (Contigen™)	Poor long-term effect
Mayer et al. (2007)	2007	Human	Calcium Hydroxylapatite (Coaptite™)	No adverse reaction
Chrouser et al. (2004)	2004	Human	Carbon Coated Beads (Durasphere™)	Poor long-term effect
Kaufman et al. (1984)	1984	Human	Polytetrafluoroethylene (Teflon™)	Highly inert materials with excellent chemical resistance, heat resistance, and low friction coefficient
Serati et al. (2019)	2023	Human	Polydimethylsiloxane (Urolastic™) Cross-Linked Polydimethylsiloxane (Macroplastique™)	Safe, effective, and minor complications
Vardar et al. (2019)	2019	Rabbit	Pig collagen Protein (Permacol™)	Bulk stability and capacity to induce muscle regeneration
Lai et al. (2017)	2017	Rabbit	Biomimetic Synthetic materials (Regensling™)	Good compliance, milder inflammatory response, stable strength
Sokol et al. (2014)	2014	Human	Polyacrylamide Hydrogel (Bulkamid^®^, Aquamid™)	Beneficial, Long-lasting

## 6 Tissue-engineered repair materials (TERMs)

The application of stem cells in tissue engineering and repair materials constitutes a significant advancement in the field of regenerative medicine, largely thanks to the unique properties of stem cells, such as their ability for self-renewal and potential to differentiate into various cell types. The primary sources of stem cells include embryos, fetuses, and adult tissues, as well as somatic cells that have been reprogrammed through genetic modification to differentiate. These are known as induced pluripotent stem cells (iPSCs) (Bacakova et al., 2018). In tissue engineering, stem cells can enhance tissue recovery by either directly integrating with and replacing damaged tissues (through differentiation) or by secreting factors that regulate the host’s response (via paracrine signaling) (Hakim et al., 2015). Table 4 summarizes the regenerative materials used to treat SUI.

**TABLE 4:** Regenerative materials for SUI.

Material	Advantages	Disadvantages
Stem cell therapy		
Urine-derived Stem Cells, (Kibschull et al., 2023)	Potential for regenerative applications, showcase muscle-specific protein expression	Technical complexity, variability in patient response
Adipose-derived Stem Cells, (Knoll et al., 2024)	Potential for significant recovery of urethral sphincter function, promotes leukocyte infiltration	Variability in efficacy, potential for immune response
Bone Marrow-Derived Stem Cells, (Sadeghi et al., 2020; Jin et al., 2016a)	Alters tissue response to acute injury, improves tissue function	Requires directed differentiation and sustained release
Dental Pulp-Derived Stem Cells, (Zordani et al., 2019)	Effective integration and restoration of urethral sphincter thickness, promotes blood vessel formation	Potential for immune response, requires precise delivery methods
Stem Cell Delivery System, (Wang et al., 2020a)	Improved cell retention, enhanced treatment efficacy	Technical complexity, potential for variability in patient response
Extracellular Components		
Extracellular Matrix Fragments, (Wang et al., 2020b)	Promotes smooth muscle tissue formation, minimally invasive	Potential integration issues, variability in patient response
Exosomes, (Liu et al., 2018) (Rolland et al., 2022) (Zhou et al., 2021)	Promotes proliferation and differentiation of skeletal muscle cells, enhances myoblast proliferation	Short half-life, requires continuous release systems
Other Materials		
Platelet-rich Plasma, (Lee et al., 2022)	Rich in growth factors, promotes tissue repair and rejuvenation	Variability in efficacy, potential for immune response
Mesoangioblasts, (Mori da Cunha et al., 2023)	Improves recovery of urethral and vaginal function, promotes nerve regeneration	Technical complexity, potential for variability in patient response
Stromal Vascular Fraction, (Boissier et al., 2016) (Inoue et al., 2018)	Aids anatomical and functional recovery of the urethra	Potential for immune response, requires precise delivery methods
Autologous Muscle Tissue, (Gräs et al., 2014)	Safe, straightforward surgical method, effective for uncomplicated SUI	Limited to straightforward cases, potential for variability in patient response
MicroRNA-29a-3p Inhibition, (Jin et al., 2016b)	Enhances outcomes of urodynamic tests, promotes collagen and elastin production	Requires co-injection with other agents, technical complexity
Autologous Skeletal Muscle Precursor Cells, (Williams et al., 2017; Williams et al., 2016b)	Effective in treating urinary incontinence, promotes muscle regeneration	Reduced effectiveness in older monkeys, those with higher body weights, and under psychosocial stress
Beta-Chitin Injectable Hydrogel, (Yang et al., 2023)	Provides urethral support, promotes stem cell homing and differentiation	Technical complexity, potential for variability in patient response

### 6.1 Stem cell therapy

#### 6.1.1 Urine-derived stem cells

**FIGURE 6:**
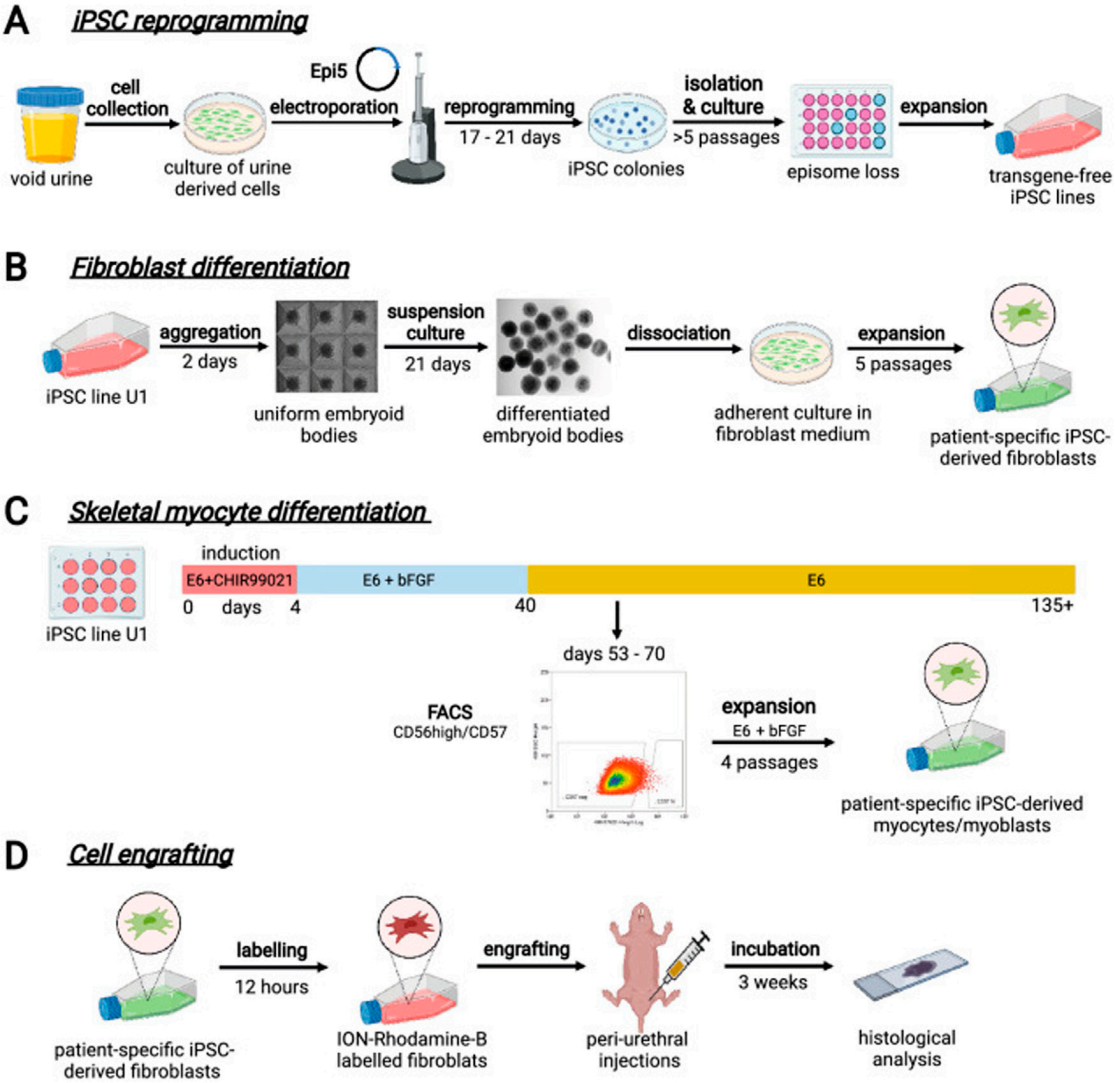
Schematic diagram of urine-derived stem cell therapy for SUI. **(A)** Reprogramming iPSCs from urine samples. **(B)** Fibroblast differentiation. **(C)** Skeletal myocyte differentiation. **(D)** Engraftment of cells into rat tissues followed by histological evaluation. Reprinted with permission from ref. (Kibschull et al., 2023). Copyright 2023, Springer Nature.

Cells from voided urine of women were reprogrammed into iPSCs. These iPSCs line U1 and hESC line H9 were differentiated into fibroblasts expressing specific markers. Myogenic differentiation was induced by CHIR99021 and confirmed by the expression of myogenic factors. Human iPSC-derived fibroblasts and myocytes were engrafted into the periurethral region of RNU rats, with successful tracking using ferric nanoparticles. This method allows scalable derivation, culture, and *in vivo* tracing of patient-specific cells to regenerate urethral damage and restore continence in rat SUI models. The research schematic is in Figure 6.

#### 6.1.2 Adipose-derived stem cells

The great potential of murine ADSC for the regeneration of urinary sphincter function has been reported. Significant and complete recovery of urethral sphincter function was observed after injection of ADSC, triggering stronger CD45^+^ leukocyte infiltration compared with the control group (Knoll et al., 2024).

#### 6.1.3 Bone marrow derived stem cells

This result has also been confirmed in human MSDC cells. Human mesenchymal stem cells can alter tissue response to acute injury and improve tissue function (Sadeghi et al., 2020). To achieve directed differentiation and sustained release of stem cells, the researchers transduced elastin into bone marrow mesenchymal stem cells (BMSCs) and used bFGF to differentiate the elastin-expressing BMSCs into fibroblasts, which produce collagen and elastin. Achieving sustained release of bFGF by formulating it in polylactic-glycolic acid (PLGA) nanoparticles resulted in significant improvements in urodynamics in disease models following treatment (Jin et al., 2016a).

#### 6.1.4 Dental pulp-derived stem cells

Human dental pulp stem cells (DPSCs) exhibit promising therapeutic effects for treating SUI. These cells have shown the ability to differentiate into muscle-related cells in laboratory conditions. After being injected, within 4 weeks, DPSCs were effectively integrated into the external urethral sphincter, almost completely restoring its thickness. This differentiation towards muscle cells persisted in the body, promoting blood vessel formation and substantially aiding in the restoration of urinary control. Additionally, the detection of hDPSCs within nerve tissues indicates their potential role in the healing of cut nerves (Zordani et al., 2019).

#### 6.1.5 Stem cell delivery system

The long-term efficacy of using stem cells directly to participate in the repair of urinary incontinence tissue is unsatisfactory, mainly due to the retention time and quantity of stem cells after injection. Thus, researchers have developed a new cell delivery system for injection therapy using superparamagnetic iron oxide (SPIO). Simply put, by constructing magnetized cells targeted and anchored at the target site, dispersed cells can be prevented from leaking into surrounding tissues after injection, thereby improving cell retention and enhancing treatment efficacy (Wang et al., 2020a).

### 6.2 Extracellular components

#### 6.2.1 Extracellular matrix fragments

The researchers injected extracellular matrix fragments of adipose stem cell sheets (ADSC ECM) into the rat urethra, where they fully integrated with surrounding tissue within 1 week. Four weeks after transplantation, host cells regenerated within the ADSC ECM fragment-injected area. Furthermore, positive staining for myosin confirmed that new smooth muscle tissue had formed around ADSC ECM fragments. These results suggest that injection of ECM fragments may be a promising minimally invasive approach to treat SUI (Wang et al., 2020b).

#### 6.2.2 Exosomes

Exosomes are nanosized membrane vesicles secreted by cells. They are a component of the intercellular communication system, carrying diverse payloads and mediating both pathogenetic and therapeutic actions, depending on the environment and cell sources (Floriano et al., 2020). Researchers extracted urine-derived stem cells exosomes (USCs-Exo) from urine and locally injected them into the pubococcygeus muscle and surrounding areas in a rat model of urinary incontinence, leading to significant improvements in urodynamic parameters. Molecular experiments revealed that USCs Exo promotes ERK phosphorylation *in vitro* and the activation, proliferation, and myotube differentiation of rat skeletal muscle SCs (Wu et al., 2019). Adipose-derived mesenchymal stem cell-secreted extracellular vesicles have been found to regulate type I collagen metabolism in fibroblasts, treating urinary incontinence (Liu et al., 2018). Human platelet-derived extracellular vesicles have shown to enhance myoblast proliferation and restore urethral sphincter function after sustained *in vivo* release. However, their very short half-life often limits significant benefits in clinical applications. A highly stable exocrine platform enables continuous release in the bioenhanced hydrogel. In a pig model of SUI, delivering PEP bioenhanced collagen one induced functional recovery of the EUS (Rolland et al., 2022). M2 macrophage-derived exosomes (M2-EXO) have been shown to reduce inflammatory cell infiltration in the SUI model and offer therapeutic effects on damaged pubococcygeal muscles (Zhou et al., 2021). ES enhances the expression of neurotrophic factors by Schwann cells, encouraging nerve cell regeneration. Meanwhile, Schwann cell-derived extracellular vesicles can stimulate neural activity, playing a role in providing therapeutic benefits for urinary incontinence (Hu et al., 2019; Zhou et al., 2018).

### 6.3 Other tissue-engineered repair materials

Platelet-rich plasma (PRP) is a multifunctional preparation widely used in regenerative medicine (Sánchez et al., 2017). It is rich in platelets as well as a large number of cytokines, chemokines and growth factors (Mussano et al., 2016). Researchers have demonstrated that injecting PRP into the urinary sphincter to repair damaged tissue and rejuvenate aged cells (Lee et al., 2022).

Intra-arterial injection of rat mesoangioblasts (MABs) and stable Vascular Endothelial Growth Factor (VEGF)-expressing MABs, improve recovery of urethral and vaginal function following simulated vaginal delivery (SVD) (Mori da Cunha et al., 2023). An important soluble GF secreted primarily from MABs is VEGF, which has myogenic and neuroprotective effects and induces nerve regeneration (Shimizu-Motohashi and Asakura, 2014; Borselli et al., 2010; Sun et al., 2003; Park et al., 2016).

Subcutaneous fat is a viable source of stromal vascular fraction (SVF) and is considered a source of autologous progenitor cells for tissue engineering and regenerative medicine applications. In a preclinical study using a large animal (pig) model of urethral injury, it was found that SVF obtained from autologous fat aids in the anatomical and functional recovery of the damaged urethra (Boissier et al., 2016). Similar results were also verified in a rat urinary incontinence model (Inoue et al., 2018).

The intraurethral administration of finely chopped autologous muscle tissue presents a straightforward surgical method that safe and fairly effective for women experiencing uncomplicated SUI. This technique is favorably comparable to a more complex regenerative approach that employs muscle-derived cells expanded *in vitro* (Gräs et al., 2014).

Inhibiting MicroRNA-29a-3p led to an increase in both the expression and secretion of elastin in BMSCs cultured *in vitro*. Furthermore, when BMSCs with MicroRNA-29a-3p inhibition were co-injected with PLGA encapsulating bFGF nanoparticles into rats with pelvic floor dysfunction (PFD) *in vivo*, there was a notable enhancement in the outcomes of urodynamic tests (Jin et al., 2016b).

Autologous skeletal muscle precursor cells (skMPC) therapy has been reported to treat urinary incontinence in a monkey disease model. Interestingly, researchers found that the effectiveness of skMPC therapy diminished in older monkeys, those with higher body weights, and monkeys experiencing psychosocial stress, which is linked to decreased production of endogenous estrogen (Williams et al., 2017; Williams et al., 2016b).

**FIGURE 7:**
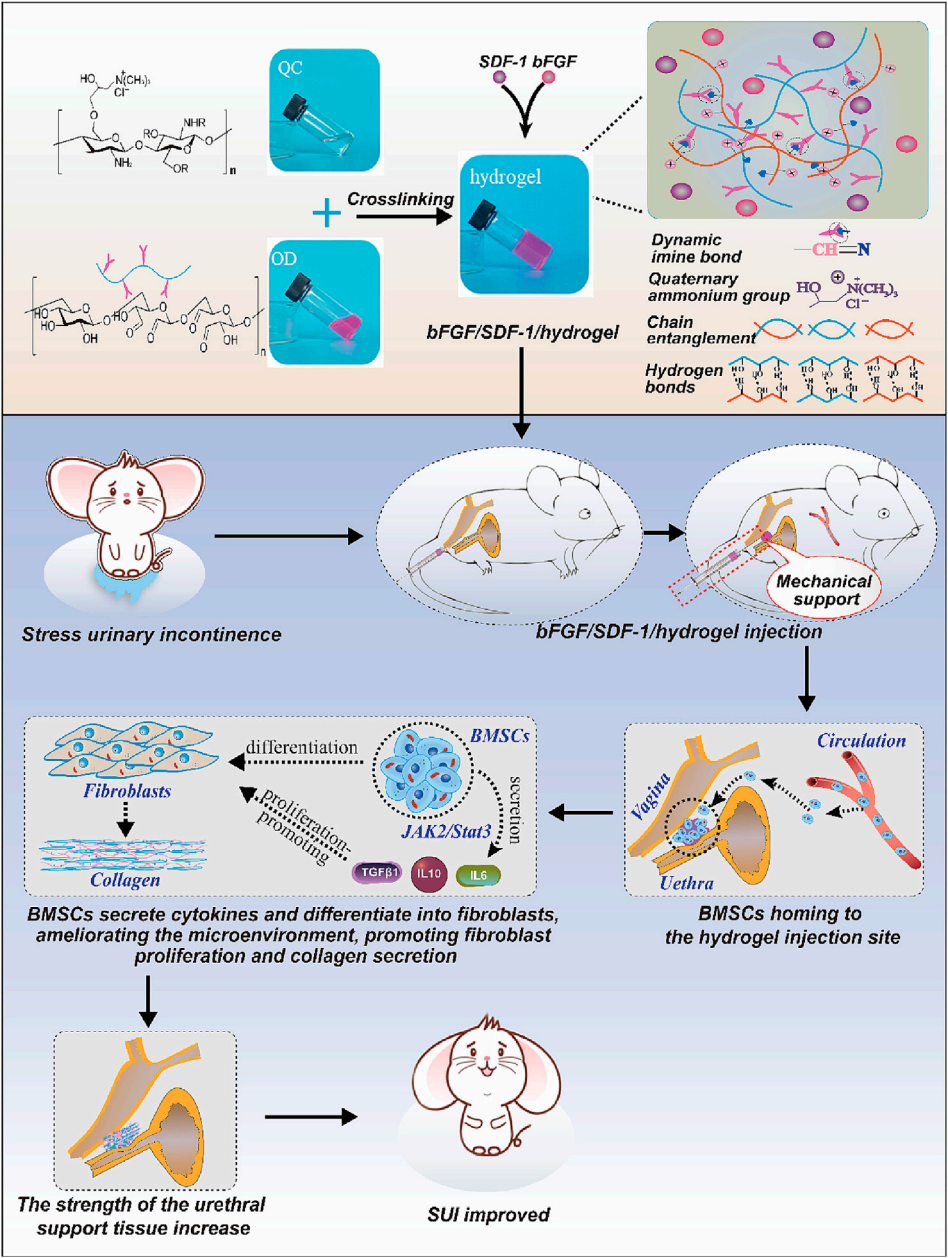
Schematic diagram of induced stem cell homing treatment for SUI. Reprinted with permission from ref. (Yang et al., 2023). Copyright 2023, Elsevier.

Inducing stem cell homing effect is also a good choice for the treatment of SUI. Researchers have developed a new injectable hydrogel based on beta-chitin (Yang et al., 2023). The rapid gel-forming properties of hydrogels with a 3% concentration can quickly stabilize SDF-1 and bFGF inside the hydrogel and delay their release *in vivo*. In the early stage, the hydrogel’s stiffness provides urethral support, similar to the TVT-O slings. In the later stage, released factors induce BMSCs to home and differentiate into fibroblasts, and the three-dimensional structure of hydrogels can stabilize BMSCs and form a cell microenvironment, which promotes fibroblast replenishment and collagen production, thereby repairing the support structure under the urethra and alleviating SUI symptoms. The system preparation and mechanism process are shown in Figure 7.

## 7 Conclusion and perspectives

This study highlights the role of both induced and spontaneous animal models in investigating SUI’s pathophysiology and the impact of innovative treatments. It delves into the potential of various biomaterials and therapeutic strategies, emphasizing the synergy between cutting-edge materials, cell therapy, and tissue engineering in addressing urinary incontinence. The findings indicate that biomaterials, such as UBAs and TERMs, along with stem cells from adipose tissue and urine, are pivotal in enhancing urethral support and regenerating sphincter function. Innovations like CAD/CAM collagen scaffolds and injectable hydrogels are making strides towards customizable, minimally invasive solutions tailored to patient needs. However, treatment variability and potential complications highlight the need for further research and therapeutic refinement. Future efforts will focus on precision in cell delivery, durability of biomaterials, and minimizing adverse effects, aiming to develop more effective and less invasive treatments for urinary incontinence, thereby enhancing patient quality of life. This outlook encourages a multidisciplinary strategy to advance urinary incontinence therapies, leveraging advancements in biomaterials, stem cell science, and tissue engineering.

## Figures and Tables

**Table:** 

**ADSC ECM**	Extracellular Matrix Fragments of Adipose Stem Cell Sheets
**BMSCS**	Bone Marrow Mesenchymal Stem Cells
**DPSC**	Dental Pulp Stem Cells
**EFS**	Electric Field Stimulation
**ERΑ**	Strogen Receptor Alpha
**ERΒ**	Estrogen Receptor Beta
**ES**	Electrical Stimulation
**EUS**	External Urethral Sphincter
**EVA**	Ethylene Vinyl Alcohol
**GAX**	Glutaraldehyde Cross-Linked
**GF**	Growth Factor
**IPSCS**	Induced Pluripotent Stem Cells
**LCE**	Iquid Crystal Elastomers
**LPP**	Leak Point Pressure
**M2-EXO**	M2 Macrophage-Derived Exosomes
**MABS**	Mesoangioblasts
**MUCP**	Maximum Urethral Closure Pressure
**MUS**	Midurethral Sling
**NDO**	Neurogenic Detrusor Overactivity
**OVX**	Ovariectomy
**PD**	Parkinson’s Disease
**PFD**	Pelvic Floor Dysfunction
**PFMT**	Pelvic Floor Muscle Training
**PLGA**	Polylactic-Glycolic Acid
**PNC**	Pudendal Nerve Crush
**PNI**	Pelvic Nerve Injury
**PNL**	Pudendal Nerve Ligation
**PNT**	Pudendal Nerve Transection
**PRP**	Platelet-Rich Plasma
**PTFE**	Polytetrafluoroethylene
**SCI**	Spinal Cord Injury
**skMPC**	Skeletal Muscle Precursor Cells
**SPIO**	Superparamagnetic Iron Oxide
**SUI**	Stress Urinary Incontinence
**SVD**	Simulated Vaginal Delivery
**SVF**	Stromal Vascular Fraction
**TERMS**	Tissue-Engineered Repair Materials
**UBAs**	Urethral Bulking Agents
**USCS-EXO**	Urine-Derived Stem Cells Exosomes
**VD**	Vaginal Distension
**VEGF**	Vascular Endothelial Growth Factor
**ZF**	Zucker Fatty
